# The hazard of mortality across different levels of frailty are increased among patients with high Braden scores

**DOI:** 10.1007/s41999-024-01062-2

**Published:** 2024-09-28

**Authors:** Hanne Nygaard, Rikke S. Kamper, Finn E. Nielsen, Sofie K. Hansen, Pernille Hansen, Miriam R. Wejse, Eckart Pressel, Jens Rasmussen, Charlotte Suetta, Anette Ekmann

**Affiliations:** 1 Department of Emergency Medicine, Copenhagen University Hospital, Bispebjerg and Frederiksberg, Copenhagen, Denmark; 2 CopenAge, Copenhagen Center for Clinical Age Research, University of Copenhagen, Copenhagen, Denmark; 3 Department of Geriatric & Palliative Medicine, Copenhagen University Hospital, Bispebjerg and Frederiksberg, Copenhagen, Denmark

**Keywords:** Clinical research, Frailty, Clinical Frailty Scale, Braden Scale, Mortality, Old age

## Abstract

**Purpose:**

To examine the prognostic accuracy of the Clinical Frailty Scale (CFS) and Braden Scale (BS) separately and combined for 90-day mortality. Furthermore, to examine the effect of frailty on mortality depending on different levels of the Braden score.

**Methods:**

The study included acutely admitted medical patients ≥ 65 years. We used an optimum cutoff for CSF and BS at ≥ 4 and ≤ 19, respectively. CFS categorized frailty as Non-frail (< 4), Frail (4–5), and Severely frail (> 5). Prognostic accuracy was estimated by the area under the receiver operating characteristic curves (AUROC) with 95% confidence intervals (CI). Cox regression analysis was used to compute the adjusted hazard ratio (aHR) for mortality.

**Results:**

The mean age among 901 patients (54% female) was 79 years. The AUROC for CFS and BS was 0.65 (CI95% 0.60–0.71) and 0.71 (CI95% 0.66–0.76), respectively. aHR for mortality of CFS ≥ 4, BS ≤ 19, and combined were 2.3 (CI95% 1.2–4.2), 1.9 (CI95% 1.3–2.9), and 1.9 (CI95% 1.3–2.8), respectively. For BS > 19, the aHR for mortality was 2.2 (CI95% 1.0–4.8) and 3.5 (CI95% 1.4–8.6) for ‘frail’ and ‘severely frail’, respectively. aHR for BS ≤ 19 was 1.1 (CI95% 0.4–3.2) and 1.3 (CI95% 0.5–3.7) for ‘frail’ and ‘severely frail’, respectively.

**Conclusion:**

Although CFS and BS were associated with 90-day mortality among older acutely admitted medical patients, the prognostic accuracy was poor-to-moderate, and the combination of CFS and BS did not improve the prognostic accuracy. However, the hazard of mortality across different levels of frailty groups were particularly increased among patients with high BS scores.

**Graphical abstract:**

## Key summary points

**Aim:**

To examine the prognostic accuracy of the Clinical Frailty Scale (CFS) and Braden Scale (BS) separately and combined for 90-day mortality.

**Findings:**

CFS and BS were associated with 90-day mortality among older acutely admitted medical patients, the prognostic accuracy was poor-to-moderate, and the combination of CFS and BS did not improve the prognostic accuracy. However, the hazard of mortality across different levels of frailty groups was particularly increased among patients with high BS scores.

**Message:**

When frailty is used in risk stratification of older acutely admitted medical patients, the frail patients’ risk of death is, to some extent, also dependent on the BS status.

## Graphical abstract

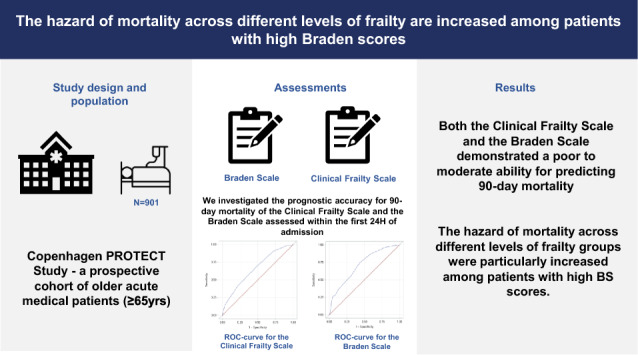

## Introduction

There is increasing recognition of the importance of frailty in clinical settings and research [1]. Frailty is a clinical condition characterized by a decline in multiple physiological systems and reserve capacity [1–3]. When the physiological reserve capacity of older individuals reaches a critically low level, even minor stressors can result in severe complications such as decreased mobility, falls, more frequent hospitalizations, disability, and mortality [1, 2, 4].

The Clinical Frailty Scale (CFS) was initially developed as a simple, user-friendly, and time-saving version of the more comprehensive Frailty Index (FI) derived from The Canadian Heart Study on Health and Aging [5]. In the validation study, Rockwood et al. used Receiver Operating Characteristic (ROC) analysis to define a CFS score of five as the optimal cutoff value for predicting mortality [5]. Several studies have shown that frailty, assessed using the CFS, is associated with increased mortality [6–9] and, therefore, a potential candidate to be used in risk stratification of older patients. However, even though the CFS is associated with mortality [10], the prognostic ability has been shown to be moderate [11]. A study by Nygaard et al. showed the CFS to have a high sensitivity, but low specificity for prediction of mortality in acutely older admitted patients [6]. Therefore, despite the ability to identify frail patients [5], the CFS is not suitable as a single prognostic screening tool in the acute setting [6]. This underlines the need for investigating the prognostic ability of other tools that, alone or in combination with CFS may be useful in predicting mortality among older acutely admitted medical patients.

The Braden Scale (BS) is a potential candidate screening tool containing information related to frailty at the point of acute hospitalization. The BS is widely used in standard care as a validated screening tool for determining the risk of pressure ulcers [12, 13] and as such, reflects the patient’s physical ability at the point of acute hospitalization. Several studies have proposed that the BS can be used to identify patients with frailty [14–16] and have, in addition, been shown to be independently associated with mortality [17]. We have found it of interest to examine if the BS can contribute with more prognostic information than CFS and if the combination of the two scales may increase the prognostic accuracy.

Prognostic models of mortality are important in an acute clinical in-hospital setting as these models strengthen the risk stratification of the patients [18]—in this case, the older acutely admitted medical patients. Comprehensive Geriatric Assessment (CGA) carried out by multidisciplinary teams with geriatric competencies is the gold standard of treatment and care planning for older, frail patients [19]. However, healthcare professionals in acute in-hospital units do not necessarily have geriatric specialist competencies. Hence, it is necessary to develop easily usable screenings to identify high-risk older patients in the initial phase of the acute admission. This population is very heterogeneous, and the models must apply to this presumption. This is the weighted argument of letting frailty be the center of the model. Therefore, the aim of this study was to examine the prognostic accuracy of the CFS and the BS, individually and combined, for 90-day mortality among acutely admitted older medical patients. Furthermore, to examine the effect of frailty on 90-day mortality depending on different levels of the Braden score.

## Material and methods

### Setting and subjects

The study was based on data from the Copenhagen PROTECT Study [20], a prospective cohort of older medical patients ≥ 65 years of age. In total, 1071 patients were included in the cohort [21]. Because of missing measurements of the BS we excluded 170 patients for the analyses of this specific study.

The CFS and the BS were evaluated within the first 24 h of the acute admission. The BS was assessed at the bedside, and the CFS was evaluated based on observations of the patients supplemented with questions on activities of daily living, dependency of relatives or healthcare professionals, and evaluation of function and cognition.

The study participants were all acutely admitted to The Acute Medical Unit (AMU) at Copenhagen University Hospital, Bispebjerg and Frederiksberg Hospital (BFH) between November 2019 and November 2021 (clinical trials: NCT04151108). The Copenhagen PROTECT Study was planned with process enrollment of patients for 12 months consecutively to cover seasonal changes in the symptoms and diseases of the older patients. However, due to COVID-19 outbreaks, the inclusion of new patients was paused three times, in total 11 months. Consequently, the total inclusion period was 13 months, covering a whole calendar year.

Patients were eligible for inclusion if they were aged ≥ 65 years and acutely admitted because of a judged medical condition. Exclusion criteria were admission for more than 24 h when assessed for baseline data uptake, terminal illness evaluated by information and diagnosis in the healthcare journal in close collaboration with the treating doctor, inability to read or speak Danish, airborne or droplet infection requiring isolation (none of the enrolled patients were hospitalized due to SARS-COV-2), judged medically contraindicated by health personnel, temporary civil registration number, and an inability to provide informed consent for participation [20].

BFH is an acute-intake community hospital in Copenhagen with a catchment area of about 483,000 inhabitants [22]. The AMU at BFH provides treatment and care in the medical specialties of lung and infectious medicine, endocrinology, geriatric medicine, gastroenterology, and acute medicine.

### Data from electronic healthcare journals

Demographic data and information on habitual residence, medication, comorbidity, severity of disease (Early Warning Score (EWS) and C-reactive protein (CRP), length of stay, and in-hospital death were collected from the electronic health care records from the index admission [20]. Information on mortality within 90 days following the admission was obtained from the Danish Civil Registration System [23].

Habitual residence was defined as either home-dwelling or nursing home. Polypharmacy was defined as five or more prescribed medications as per protocol [20]. Comorbidity was assessed by the Charlson Comorbidity Index (CCI) [20]. The weighted index was used to describe the patient’s degree of comorbidity. A CCI score from 1–2 was interpreted as “mild”, 3–4 as “moderate”, and ≥ 5 as “severe” comorbidity [24]. Length of stay (LOS) was dichotomized in stay under and above 120 h, equaling 5 days as cutoff of short and long stay.

### Statistical analysis

The primary outcome was time to all-cause mortality (time-to-event analysis) within 90 days following admission.

Data were presented as means with standard deviation (SD) for normally distributed variables, median with interquartile range (IQR) for non-normally distributed continuous variables or counts, and percentages for categorical variables. Sensitivity, specificity, the positive predictive value (PPV), and the negative predictive value (NPV) were calculated with the corresponding 95% confidence interval (CI).

The receiver-operating characteristics (ROC) curves for the CFS and the BS were computed, and the area under the operating characteristics (AUROC) was calculated with 95% CI to assess the prognostic accuracy of the CFS and the BS. DeLong’s test [25] was performed to test if the two ROC curves differed significantly. To capture the performance of the CFS and the BS as dichotomous diagnostic tests, the Youden’s index (YI) was calculated to define the optimum cutoff values on both scales for predicting 90-day mortality [26]. The discriminative performance was based on the AUROC and ranges from 0.5 and 1.0, where a AUROC 0.7–0.8 equals acceptable performance, 0.8–0.9 excellent performance, and > 0.9 outstanding performance [27, 28].

Cox regression analyses were used to calculate hazard ratios (HR) with 95% CI for 90-day mortality for the CFS and the BS separately and combined adjusted for potential confounding. Potential confounders (sex, age, comorbidity degree, EWS, CRP, LOS, residence) with a significant association with mortality in the univariate analyses were selected for inclusion in the multivariate regression analyses. Person-days of follow-up were calculated from the date of admission to the date of death or the date at the end of follow-up (90 days), whichever came first. The HR is presented as a crude model and adjusted model. The model adjustments (aHR) were done by including all covariates followed by backward elimination and, thereby, creating a final multivariable model keeping only covariates with *p* < 0.05. The final models are specified in footnotes in connection with the tables. The proportional hazard assumption of all variables was tested and found satisfactory except for the continuous variable of length of stay. A dichotomized variable was derived guided by the median value of the continuous variable. The dichotomized variable met the proportional hazard assumption and was still highly associated with mortality, like the continuous variable of the length of stay.

Furthermore, survival probability according to the dichotomized CFS-variable combined with the BS groups (CFS < 4 and BS > 19, CFS ≥ 4 and BS > 19, CFS < 4 and BS ≤ 19, and CFS ≥ 4 and BS ≤ 19) are presented graphically using the Kaplan–Meier survival function. The survival curves were compared by the logrank test.

For the “non-frail”, the “frail”, and the “severely frail” groups we have investigated if the aHR for 90-day mortality was modified by different levels of BS. The calculated optimum cutoff point of BS from the present study was used to divide the patients into two different BS levels.

A *p*-value < 0.05 was considered statistically significant. All statistical analyses were performed in SAS Studio.

## Results

In total, 901 persons had complete information on the CFS and the BS, and were followed for a total of 3990.25 days, ranging from 0.6 to 89.9 days. Mean age was 79 years ± 7.9 (53.5% female). The 170 (15.9%) patients who had missing data on the BS, and were therefore excluded from the analysis of this study, did not differ significantly in sex, age, comorbidity, and mortality.

A total of 108 (12.0%; 95% CI 9.9–14.3) patients died within 90 days following admission, of whom 31 (3.4%; 95% CI 2.3–4.8) died during hospitalization.

Patient characteristics are presented in Table 1. In the group of patients dying within 90 days after admission we found a significantly higher proportion of men, higher age, higher degree of comorbidity, longer LOS, and increased severity of disease reflected as both a higher EWS and higher CRP. Furthermore, we found a higher proportion of patients living at nursing homes among patients who died within 90 days after admission compared to survivors (Table 1).

**Table 1:** Baseline characteristics according to survival status within 90-days after admission

Characteristics	All patients (*N* = 901)	Non-survivors (*n* = 108)	Survivors (*n* = 793)	*P* value
Sex				** < 0.01**^**a**^
Men, *n* (%)	419 (46.5)	65 (60.2)	354 (44.6)	
Women, *n* (%)	482 (53.5)	43 (39.8)	439 (55.4)	
Age, mean (SD)	79.0 (7.9)	81.1 (7.6)	78.6 (7.9)	** < 0.01**^**b**^
BMI, mean (SD)	25.9 (5.2)	24.9 (4.9)	26.0 (5.2)	0.10^b^
Comorbidity degree:				** < 0.01**^**a**^
Mild (CCI 1–2), *n* (%)	31 (3.4)	1 (0.9)	30 (3.8)	
Moderate (CCI 3–4), *n* (%)	351 (39.0)	20 (18.5)	331 (41.7)	
Severe (CCI ≥ 5), *n* (%)	519 (57.6)	87 (80.6)	432 (54.5)	
Medical specialties				0.08^a^
Geriatric medicine, *n* (%)	355 (39.4)	42 (38.2)	313 (39.6)	
Lung- and infectious medicine, *n* (%)	262 (29.1)	42 (38.2)	220 (27.8)	
Endocrinology, *n* (%)	193 (21.4)	14 (12.7)	179 (22.6)	
Gerontology, *n* (%)	89 (9.9)	12 (10.9)	77 (9.7)	
Acute medicine, *n* (%)	2 (0.2)	0 (0.0)	2 (0.3)	
Medication				
Polypharmacy, *n* (%)	723 (80.2)	87 (80.6)	636 (80.2)	0.93^a^
Number of medications, mean (SD)	8.4 (4.5)	8.8 (4.6)	8.3 (4.4)	0.28^b^
Severity of disease				
EWS, median (IQR)	2 (0; 4)	3 (1; 5)	2 (0; 4)	**0.02**^**c**^
C-reactive protein, median (IQR)	30.0 (5.0; 97.0)	51.5 (15.5; 103.5)	26.0 (4.0; 96.0)	** < 0.01**^**c**^
Length of stay, hours mean (SD)	180.2 (216.9)	283.5 (268,7)	165.9 (204.8)	** < 0.01**^**b**^
≤ 120 h, *n* (%)	455 (50.5)	33 (30.6)	422 (53.2)	** < 0.01**^**a**^
> 120 h, *n* (%)	446 (49.5)	75 (69.4)	371 (46.8)	
Habitual residence				**0.04**^**a**^
Home-dwelling, *n* (%)	825 (91.6)	95 (86.4)	730 (92.3)	
Nursing home, *n* (%)	76 (8.4)	15 (13.6)	61 (7.7)	
Frailty				** < 0.01**^**a**^
CFS < 4, *n* (%)	241 (26.8)	12 (10.9)	229 (29.0)	
CFS ≥ 4, *n* (%)	660 (73.3)	98 (89.1)	562 (71.0)	
Braden				** < 0.01**^**a**^
BS ≤ 19, *n* (%)	304 (33.7)	59 (54.6)	245 (30.9)	
BS > 19, *n* (%)	597 (66.3)	49 (45.4)	548 (69.1)	
Frailty and Braden combined				** < 0.01**^**a**^
CFS ≤ 3 and/or BS > 19, *n* (%)	623 (69.2)	53 (49.1)	570 (71.9)	
CFS ≥ 4 and BS ≤ 19, *n* (%)	278 (30.9)	74 (50.9)	303 (28.1)	

*BMI* Body Mass Index, *BS* Braden Scale, *CCI* Charlson Comorbidity Index, *CFS* Clinical Frailty Scale, *EWS* Early Warning Score, *IQR* interquartile range, *SD* Standard Deviation

^a^Chi-square test

^b^Unpaired *t*-test

^c^Nonparametric *z*-test

For the CFS, the optimum cutoff value was 4 with a YI at 19.8. The specificity, sensitivity, PPV, and NPV for a CFS score ≥ 4 were 88.9 (83.0–94.8), 28.9 (25.7–32.0), 14.6 (11.9–17.2), and 95.0 (92.3–97.8), respectively (Table 2). For the BS, the optimum cutoff was 19 by YI at 30.4. The specificity, sensitivity, PPV, and NPV for a BS score ≤ 19 were 69.1 (65.9–72.3), 54.6 (45.2–64.0), 19.4 (15.0–23.9), and 91.8 (89.6–94.0), respectively (Table 2). Among patients fulfilling both CFS ≥ 4 and the BS ≤ 19, we found a specificity, sensitivity, PPV, and NPV of 72.9 (68.8–75.0), 50.9 (41.5–60.4), 19.8 (15.1–24.5), and 91.5 (89.3–93.7), respectively (Table 2).

**Table 2:** Sensitivity, specificity, NPV, PPV, and HR for the CFS and the BS individually and combined in relation to 90-day mortality

	Specificity (95% CI)	Sensitivity (95% CI)	PPV (95% CI)	NPV (95% CI)	HR^a^ (95% CI)	aHR^b^ (95% CI)	aHR (95% CI)
CFS ≥ 4							
*N* = 660 (73.3%)	88.9 (83.0–94.8)	28.9 (25.7–32.0)	14.6 (11.9–17.2)	95.0 (92.3–97.8)	3.1 (1.7–5.6)	2.1 (1.1–3.9)	2.3^c^ (1.2–4.2)
BS ≤ 19							
*N* = 304 (33.7%)	69.1 (65.9–72.3)	54.6 (45.2–64.0)	19.4 (15.0–23.9)	91.8 (89.6–94.0)	2.6 (1.7–3.7)	1.9 (1.2–2.8)	1.9^d^ (1.3–2.9)
CFS ≥ 4 and BS ≤ 19							
*N* = 278 (30.9%)	72.9 (68.8–75.0)	50.9 (41.5–60.4)	19.8 (15.1–24.5)	91.5 (89.3–93.7)	2.5 (1.7–3.6)	1.8 (1.2–2.7)	1.9^d^ (1.3–2.8)

*aHR* adjusted hazard ratio, *BS* Braden Scale, *CFS* Clinical Frailty Scale, *EWS* Early warning score, *HR* hazard ratio, *NPV* negative predictive value, *PPV* positive predictive value

^a^HR: crude hazard ratio

^b^aHR: hazard ratio adjusted for age, sex, comorbidity degree, EWS, C-reactive protein, length of stay, and habitual residence

^c^aHR: hazard ratio adjusted for sex comorbidity degree, EWS, and length of stay

^d^aHR: hazard ratio adjusted for age sex comorbidity degree, EWS, and length of stay

A total of 660 (73.3%) patients had a CFS score ≥ 4. For BS, 304 (33.7%) patients scored ≤ 19. In total, 278 (30.9%) patients passed the optimum cutoff points for both screening tools.

**Fig. 1:**
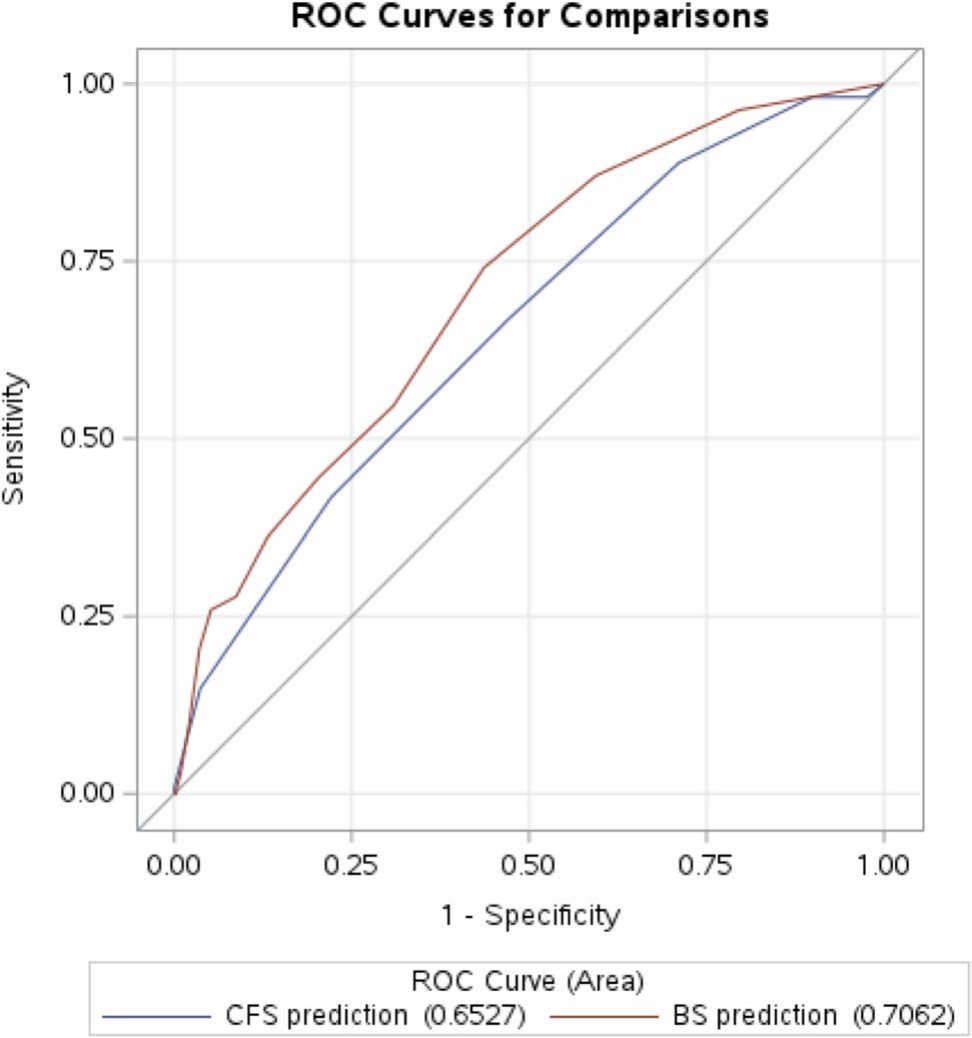
Receiver operating characteristics curves for the Clinical Frailty Scale (blue) and for the Braden Sciale (red)

aHR for 90-day mortality of the CFS ≥ 4, BS ≤ 19, and fulfilling both CFS ≥ 4 and BS ≤ 19 were 2.3 (1.2–4.2), 1.9 (1.3–2.9), and 1.9 (1.3–2.8), respectively (Table 2). The ROC-curves for CFS and BS are presented in Fig. 1. The AUROC for CFS and BS was 0.65 (0.60–0.71) and 0.71 (0.66–0.76), respectively, with no statistically significant difference between the two curves (*p* = 0.05) (Fig. 1).

**Fig. 2:**
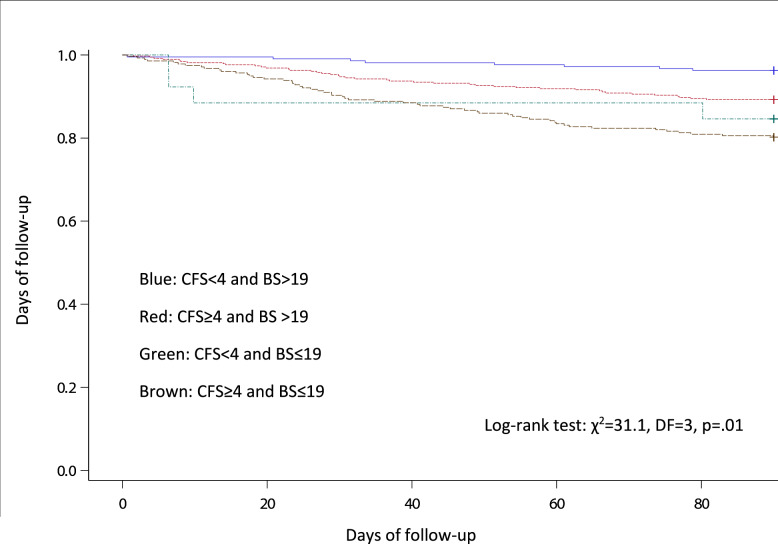
Kaplan–Meier Survival Function of the association between the combination of CFS and BS and 90-day mortality

Figure 2 shows the Kaplan–Meier survival function for patients with none, one, or both CFS ≥ 4 and BS ≤ 19 during the 90 days of follow-up (*p* < 0.01). The cases and proportions of non-survivors according to the combination of different levels of CFS and BS are reported in Table 3. Table 4 shows frailty grouped in three levels and stratified by the level of BS. For BS > 19 we found an aHR for 90-day mortality at 2.2 (1.0–4.8) and 3.5 (1.4–8.6) for the frail and the severely frail, respectively. In comparison, the aHR for BS ≤ 19 was 1.1 (0.4–3.2) and 1.3 (0.5–3.7) for the frail and the severely frail, respectively.

**Table 3:** Number of patients and 90-day mortality in each level of frailty and stratified by BS

	*N*	90-day mortality, *n* (%)
Non-frail and a BS > 19	215	8 (3.7)
Non-frail and a BS ≤ 19	26	4 (15.4)
Frail and a BS > 19	313	29 (9.3)
Frail and a BS ≤ 19	127	22 (17.3)
Severely frail and a BS > 19	69	12 (17.4)
Severely frail and a BS ≤ 19	151	33 (22.4)

*BS* Braden Scale

**Table 4:** Hazard ratios for 90-day mortality on each level of frailty stratified by BS

	*N*	90-days mortality, *n* (%)	aHR (95% CI)^a^ BS > 19 (*n* = 597)	aHR (95% CI)^b^ BS ≤ 19 (*n* = 304)
Non-frail	241	12 (5.0)	Ref	Ref
Frail	440	51 (11.6)	2.2 (1.0–4.8)	1.1 (0.4–3.2)
Severely frail	220	5 (20.5)	3.5 (1.4–8.6)	1.3 (0.5–3.7)

*aHR* adjusted hazard ratio, *BS* Braden Scale, *CI* Confidence Interval

^a^The model was adjusted for comorbidity degree and length of stay

^b^The model was adjusted for sex, comorbidity degree, and length of stay

## Discussion

Our study revealed that the discriminative performance of the BS for predicting mortality was slightly better than the CFS. However, the difference was not significant, and the overall performance of both scores was poor to moderate. The combined model of CFS and BS did not improve the prognostic accuracy of 90-day mortality. However, an important finding from this study was that the hazard of mortality across the different levels of frailty was primarily increased among patients with BS scores above 19 compared to patients with lower BS scores.

Our study is the first to define an optimum cutoff value for the BS for predicting 90-day mortality following admission in older acutely admitted medical patients. In the association between the BS and mortality, we found a twofold increase in the risk of 90-day mortality by the estimated cutoff point of 19. Of note, the Kaplan–Meier plot shows few non-survivors in the group of CFS < 4 and BS ≤ 19. However, these non-survivors die within the first 10 days after admission compared to death in the other groups, which appears throughout the follow-up period. In line with our results, previous studies have found low BS scores associated to both in-hospital and all-cause mortality [15, 17, 29, 30]. These studies applied the BS as a continuous scale [15, 17], or the originally defined cutoffs of the BS used for risk stratification of pressure ulceration [29, 30]. However, using the BS as a continuous scale is clinically impractical. The purpose of defining the optimum cutoff point for the BS was to increase the ease of use in the clinical setting. Notably, the AUROC for the BS was 0.71 interpreted as a moderate result of the overall prognostic accuracy.

The CGA is a multidimensional process based on an interdisciplinary approach to identify and manage frailty strongly associated with adverse outcomes such as mortality [19]. However, completing a full CGA is time-consuming, and CGA is not fully applicable in the acute wards as the specialized multidisciplinary resources are not allocated here. Hence, the need for CGA could be qualified by a routinely performed frailty screening [31]. Our study evaluated the CFS assessed in the AMU, where this screening could act as a potential and alternative risk stratifier for older acutely admitted medical patients.

Our findings of frailty were consistent with those from earlier studies regarding the association with all-cause mortality [6–9, 11, 32]. This result emphasizes the concept of frailty as a clinical condition where the older individuals have low physiological reserve capacity [1, 2]. However, adding to these findings, our study also showed that the CFS had a poor ability to predict mortality when assessed during acute hospitalization, with an AUROC of 0.65 similar to the results from Belga et al. [11]. Notably, we found the optimum cutoff to be 4, which differs from the cutoff point of 5 estimated in the validation study by Rockwood et al. [5]. This discrepancy could be caused by variations in the groups of included patients. Our exclusion of terminally ill patients could have caused a decrease in patients scoring 8–9 on the CFS. Other than that, using CFS in the acute setting increases the risk of measurement bias leading to misclassification due to the retrospective perspective used in the CFS assessment. Furthermore, since the evaluation of CFS reflects the period towards the acute admission, a potential exacerbation of chronic illness, poor recall, and stressors in the emergency department environment could lead to incorrect classification of the level of frailty [33].

To our knowledge, the effect of frailty on 90-day mortality according to different levels of the BS has never been investigated. A study by Jung and colleagues [34] found an increased CFS was highly associated with an increased risk of pressure ulcers evaluated by BS and discussed that the CFS might be useful as a universal predictor of geriatric outcomes, including pressure ulcerations. In the results of our stratified analysis, we also found an association between the BS and the CFS. Additionally, we found that having a score of BS ≤ 19 negatively modified the effect of frailty at all levels and, thus, found the highest risk of mortality among frail patients with a BS score > 19. Hence, the low score on the BS, interpreted as decreased physical ability at the point of acute hospitalization, attenuated the otherwise significant impact of frailty on mortality. Frail older acutely admitted patients with a BS score > 19 may have a high CFS category due to multimorbidity not limiting mobility. From this perspective, assessment of the BS in combination with the CFS adds valuable information and captures different high-risk patient groups. In contrast to Jung et al. [34], our findings advocate the assessment of both screenings. Adding BS score in an overall evaluation of frailty may be helpful in the risk stratification of older acutely admitted medical patients and is, therefore, suggested to be included and tested in future prediction models.

As the hospitalization itself represents a stressor that increases the risk of physical deconditioning for the older patients [35] initial evaluation must be of highest prioritization. This finding underlines that even minor disabilities related to nutrition, mobility, physical function, and activity represent a major risk for the older medical patient.

### Strengths and limitations

The major strength of the present study was the prospective design and that the CFS and the BS were assessed within the first 24 h following admission without any knowledge to the patients’ outcome. The assessment of the CFS and the BS within the first 24 h of admission elaborated the advantages and disadvantages of using the CFS and the BS in the acute clinical setting. Additionally, the consecutive inclusion with only few exclusion criteria minimized the risk of selection bias. Likewise, it was a strength that all data from the medical records were collected by healthcare employees in the project and controlled independently by another (HN).

A limitation is the exclusion of terminally ill patients. Due to the investigation of the CFS, this specific exclusion criterion may have affected the prevalence of the most frail patients in the study.

Additionally, it is a limitation and potential risk of selection bias that the Copenhagen PROTECT Study was conducted during the COVID-pandemic. The enrollment was paused during periods with a high prevalence of the virus, but the presence of patients in the AMU with suspected COVID infection was persistent during the second part of 2020 and throughout 2021. With reference to the exclusion criteria patients isolated with suspected or verified COVID-19 infection were excluded if the isolation exceeded more than 24 h from the time of admission. Another limitation is the study being a single-center study. Likewise, the sample size of this study is not based on a power calculation but is determined by the number of patients with complete information on the CFS and the BS in the PROTECT Study [20]. The estimates in the analyses of the impact of frailty on mortality in different BS groups may be imprecise due to the low rates of mortality in some of the groups. It is possible that a larger sample size could have increased the precision of these and other estimates in our study. Last, residual confounding may be present. An example is nutritional status for which we do not have sufficient information. The missing information on nutrition might cause an overestimation of the association between CFS and mortality. However, nutritional status is a domain of the BS, and hence, confounder control may cause over-adjusting and underestimation of the risk of BS. Another example of a covariate not included is habitual functional status. Lack of adjustment may cause residual confounding and overestimation of the association between BS and mortality but underestimation of the association between CFS and mortality. In general, confounder control has to be carried out with caution when investigating the association between syndromes and various outcomes, as the risk of underestimation is substantial due to the complex composition of the syndromes.

### Clinical implications

The population of older medical patients is heterogeneous regarding deconditioning, disabilities, multimorbidity, and cognitive impairment [36, 37], which highlights the need for prognostic models of adverse outcomes with high accuracy regardless of this presumption. The differences and heterogeneity in the older medical patients clarified a need for measurements of frailty, and the CFS is increasingly implemented in both Emergency Departments and medical wards. The CFS is preferred over other frailty measures because of its clinical usability, low time consumption, and ease of use [6, 38].

This study provides new knowledge on whether measures of frailty can be considered as indicators to be used in risk stratification of older acutely admitted medical patients. The analysis of the combined score of CFS ≥ 4 and BS ≤ 19 did not improve the prognostic ability of the scores. However, the findings in our study suggest that frail patients’ risk of death is, to some extent, also dependent on the BS status. This underlines the importance of performing both measures of BS and CFS as these scales provide important and different information regarding risk stratification and mortality in acutely admitted older medical patients. However, the findings in our study should be confirmed and tested in future prediction studies.

## Conclusion

Older acute medical patients identified as frail are at greater risk of dying within 90 days. Although both CFS and BS were associated with mortality, the prognostic accuracy was poor-to-moderate. The combination of CFS and BS did not improve the prognostic accuracy. However, the hazard of mortality across the different frailty groups were increased among patients with a BS score > 19 compared to patients with a lower BS score. We suggest that these findings should be included and tested in future prediction models.

## Data Availability

The data used in the analysis for the present study are available from the corresponding author [HN] upon reasonable request.
